# Validity and Reliability of Facial Rating of Perceived Exertion Scales for Training Load Monitoring

**DOI:** 10.1519/JSC.0000000000004361

**Published:** 2023-03-22

**Authors:** Stephan van der Zwaard, Folef Hooft Graafland, Cerianne van Middelkoop, Lotte L. Lintmeijer

**Affiliations:** 1 Leiden Institute of Advanced Computer Science, Leiden University, Leiden, The Netherlands;; 2 Department of Human Movement Sciences, Vrije Universiteit Amsterdam, Amsterdam Movement Sciences, Amsterdam, The Netherlands;; 3 Fitchannel.com, Amsterdam, The Netherlands; and; 4 Department of Applied Mathematics, Technical University Delft, Delft, The Netherlands

**Keywords:** home-based exercise, training monitoring, RPE, criterion validity, user experience

## Abstract

van der Zwaard, S, Hooft Graafland, F, van Middelkoop, C, and Lintmeijer, LL. Validity and reliability of facial rating of perceived exertion scales for training load monitoring. *J Strength Cond Res* 37(5): e317–e324, 2023—Rating of perceived exertion (RPE) is often used by coaches and athletes to indicate exercise intensity, which facilitates training load monitoring and prescription. Although RPE is typically measured using the Borg’s category-ratio 10-point scale (CR10), digital sports platforms have recently started to incorporate facial RPE scales, which potentially have a better user experience. The aim of this study was to evaluate the validity and reliability of a 5-point facial RPE scale (FCR5) and a 10-point facial RPE scale (FCR10), using the CR10 as a golden standard and to assess their use for training load monitoring. Forty-nine subjects were grouped into 17 untrained (UT), 19 recreationally trained (RT), and 13 trained (T) individuals Subjects completed 9 randomly ordered home-based workout sessions (3 intensities × 3 RPE scales) on the Fitchannel.com platform. Heart rate was monitored throughout the workouts. Subjects performed 3 additional workouts to assess reliability. Validity and reliability of both facial RPE scales were low in UT subjects (intraclass correlation [ICC] ≤ 0.44, *p* ≤ 0.06 and ICC ≤ 0.43, *p* ≥ 0.09). In RT and T subjects, validity was moderate for FCR5 (ICC ≥ 0.72, *p* < 0.001) and good for FCR10 (ICC ≥ 0.80, *p* < 0.001). Reliability for these groups was rather poor for FCR5 (ICC = 0.51, *p* = 0.006) and moderate for FCR10 (ICC = 0.74, *p* < 0.001), but it was excellent for CR10 (ICC = 0.92, *p* < 0.001). In RT and T subjects, session RPE scores were also strongly related to Edward's training impulse scores (*r* ≥ 0.70, *p* < 0.001). User experience was best supported by the FCR10 scale. In conclusion, researchers, coaches, strength and conditioning professionals, and digital sports platforms are encouraged to incorporate the valid and reliable FCR10 and not FCR5 to assess perceived exertion and internal training load of recreationally trained and trained individuals.

## Introduction

Athletes and coaches have incorporated the rating of perceived exertion (RPE) to estimate the intensity of their workouts (14). The RPE is relatively simple to use: just asking athletes “how was your workout?” while capturing their answer in a single score. Typically, RPE scores are obtained using the Borg’s 6–20 RPE scale (3) or the modified Borg’s category-ratio 10-point scale (CR10 (14)), which can be used interchangeably (1). Besides its simplicity, the session RPE (i.e., RPE based on CR10 × duration) is considered to be a very useful tool for monitoring internal training load because it reflects periodization of internal training load as well as positive and negative training outcomes (15). Additional information can be derived from complementary indices that are calculated from session RPE scores, such as monotony and strain, reflecting day-to-day training variability and total stress on the body (17). Moreover, RPE and session-RPE using the CR10 have shown to be valid and reliable (17,18), which makes the CR10 a golden standard for perceived exertion.

Recently, digital sport platforms (13,25–28) have started to incorporate various RPE scales to assist healthy individuals up to professional athletes with monitoring their internal training load in a real-life setting. For those platforms, facial RPE scales that are supported by emoticons are of particular interest because these are easier to understand and may have a better user experience (5,21). Facial RPE scales with limited options are likely most appealing because these can be easily used on mobile devices. However, before digital sport platforms can confidently use these facial RPE scales for training monitoring, their validity and reliability should first be verified, preferably in a real-life setting with high ecological validity. Few studies have evaluated the criterion validity of facial RPE scales, demonstrating high validity, but only in well-controlled laboratory-based settings and not in the context of training load monitoring (5,21,22). It remains to be determined whether facial RPE scales can be used interchangeably with the CR10 to quantify the internal training load of workouts in a real-life setting.

The aim of this study was to evaluate the validity and reliability of a 5-point facial RPE scale (FCR5) and a 10-point facial RPE scale (FCR10) in a real-life setting, using the CR10 as a golden standard. In addition, we compare the associations between Edward’s training impulse scores and session RPE values calculated using the facial RPE scales to the association for the CR10 scale. This comparison provides an indication of the validity of facial RPE scales for internal training load monitoring. Based on previous findings (5,21,22), it was hypothesized that FCR5 and FCR10 reveal good validity. Accordingly, we expected good reliability of these facial RPE scales and that FCR5 and FCR10 can be used interchangeably with CR10 to quantify the internal training load of a workout.

## Methods

### Experimental Approach to the Problem

To assess baseline physical fitness, all subjects completed a maximal incremental exercise test in the first week of this validation study. After the first week, subjects performed a home-based training program consisting of 12 workout sessions divided over 6–7 weeks. After every workout session, perceived exertion was evaluated using one of the 3 RPE scales (i.e., FCR10, FCR5, or CR10 (14)). Rating of perceived exertion scores from the first 9 workout sessions were used to validate the FCR5 and FCR10 scale at 3 intensities, using CR10 as a golden standard. Validity was determined based on absolute agreement of RPE scores and the relationship with average heart rate. Rating of perceived exertion scores from the last 3 workout sessions were used to examine the reliability of the facial scales, and it served as retest for one of the RPE scales at the 3 intensities. Moreover, associations between session RPE and Edward's TRIMP were based on all 12 workout sessions. Subjects were instructed to avoid strenuous exercise within the last 24 hours before the incremental test and home-based workouts.

### Subjects

Sixty-one healthy individuals (44 women and 17 men) participated in the study. Inclusion criteria were as follows: (a) healthy individuals between 18 and 55 years and (b) without injuries or chronic health conditions (e.g., severe asthma, diabetes, or heart disease). Forty-nine subjects (age range: 22–54) were included in the analysis, as 12 individuals dropped out before completing the first 9 validation workouts. Based on the individual peak power output (W·kg^−1^) during the incremental exercise test, subjects were grouped into 17 untrained (UT), 19 recreationally trained (RT), and 13 trained (T) groups (7,8). Subject characteristics are displayed in Table 1. Before the study, subjects were informed about the aim and the protocol of the study, after which they provided written informed consent. The study was conducted based on principles articulated in the Declaration of Helsinki and was approved by the Departmental Ethics Committee of the Vrije Universiteit, Amsterdam, The Netherlands (VCWE-2021-051).

**Table 1:** Subject characteristics.*

	Untrained	Recreationally-trained	Trained
N	17	19	13
Females | males	14 | 3	12 | 7	8 | 5
Age (y)	38 ± 8.4	39 ± 9.2	34 ± 9.7
Body mass (kg)	81 ± 15.3	73 ± 12.5	72.4 ± 9.8
POpeak (W)	210 ± 55.2	277 ± 66.9†	320 ± 61.2†
POpeak (W/kg)	2.6 ± 0.5	3.8 ± 0.4†	4.4 ± 0.4†‡
HRpeak (bpm)	182 ± 11	182 ± 9	187 ± 8
Graded exercise time (s)	765 ± 137.2	813 ± 103.6	815 ± 69.8

* HR = heart rate, PO = power output.

† Significantly different from untrained subjects.

‡ Significantly different from recreationally-trained subjects.

### Procedures

#### Rating of Perceived Exertion Scales

**Figure 1.**
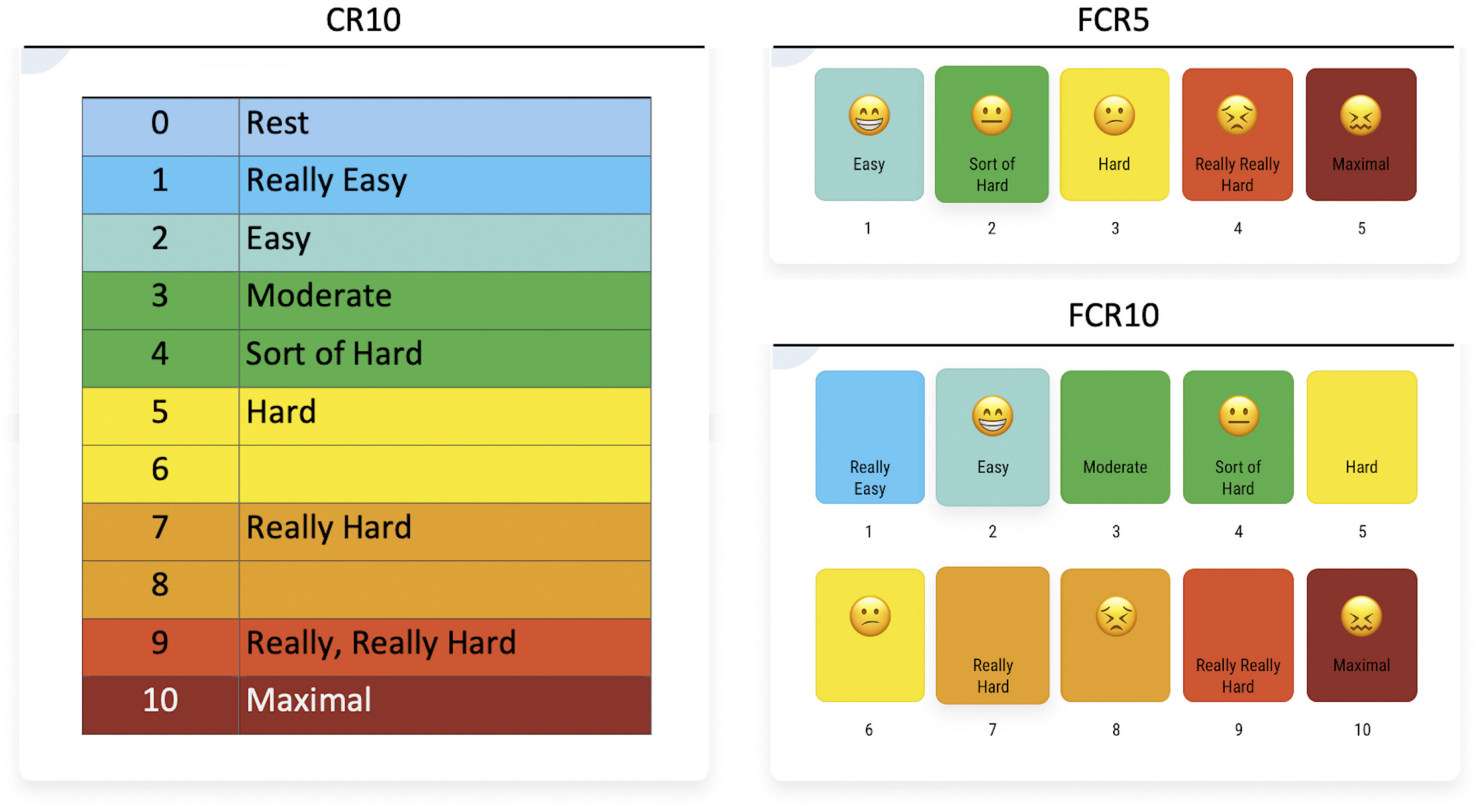
Rating of perceived exertion scales that were validated and tested for their reliability: the 10-point category-ratio RPE scale (CR10), a 5-point facial RPE scale (FCR5), and a 10-point facial RPE scale (FCR10). Note that Dutch translations of the verbal anchors were used in this study to improve subjects' comprehension of the RPE scales. RPE = rating of perceived exertion.

Perceived exertion was evaluated after every workout session. Subjects were asked “How was your workout?” and were instructed to give a global rating of perceived exertion for the entire session, using one of the 3 RPE scales (i.e., FCR10, FCR5, or CR10; Figure 1). The CR10 refers to the commonly used Borg’s category-ratio 10-point scale (14), including explanatory phrases for the RPE scores. The facial CR10 (FCR10) is the same as the CR10, but with the addition of facial expressions (i.e., smileys) at the RPE scores of 2, 4, 6, 8, and 10. The FCR5 includes only 5 RPE scores, supported by both explanatory phrases and facial expressions (i.e., smileys). User experience with the RPE scales was also evaluated at the end of the study using a short questionnaire. This questionnaire included questions regarding which of the RPE scales the subjects favored, whether they preferred 5-point or 10-point scales and whether they preferred RPE scales supported by smileys (i.e., facial expressions) or without smileys.

#### Incremental Exercise Test

In the first week, subjects performed a maximal incremental ramp test to voluntary exhaustion on an electromagnetically braked cycle ergometer (Excalibur or Excalibur Sport, Lode, Groningen, The Netherlands). After pedaling at 0 W for one minute, power output was increased continuously by 15–30 W per minute, depending on the subject's gender and self-reported training history (UT males: 20 W·min^−1^, RT males: 25 W·min^−1^, T males: 30 W·min^−1^; UT females: 15 W·min^−1^, RT females: 20 W·min^−1^, T females: 25 W·min^−1^). During the test, heart rate was continuously monitored using a heart rate sensor chest strap (H9, Polar Electro Oy, Finland). Heart rate (HR) data were downloaded after exercise using the online Polar software (Polar FlowSync 5.5.0, Polar Electro 2021) and Sport Data Valley-platform (26). After familiarization, subjects reported their RPE score every minute of the incremental exercise using the CR10 scale. The test was terminated if subjects could not maintain a pedaling speed above 60 rpm, despite verbal encouragement. Peak power output and peak heart rate were obtained from the test.

#### Home-Based Workouts

After the first week, validity and reliability of the facial RPE scales was determined in a real-life setting during home-based workout sessions, to ensure a high ecological validity. Workout videos were selected on the Fitchannel platform (13), reflecting exercises at a low, medium, and high intensity for each group (i.e., UT, RT, and T subjects). The videos contained full-body workouts of 29 ± 6 minutes with multiple exercises, such as squats, lunges, planking, push-ups, crunches, jumping jacks, burpees, mountain climbers, or boxing, while exercising at home. Heart rate was monitored throughout the workout using a heart rate sensor chest strap (H9, Polar Electro Oy, Finland). All subjects performed low- (L), medium- (M), and high (H)-intensity workouts for each of the 3 RPE scales during the first 9 workout sessions (3 intensities × 3 RPE scales). To examine reliability of the different RPE scales, subjects performed another 3 workout sessions for one of the 3 RPE scales (3 intensities × 1 RPE scale). Intensities were shuffled in a fixed order for all subjects (L-M-L-M-H-L-H-M-H-L-M-H). RPE scales after each session were provided in a randomized order, by allocating subjects to one of 3 randomization sequences. This random sequence determined which RPE scale was used after each workout session, and which RPE scale was retested during the final 3 sessions. Reliability was assessed by comparing RPE scores of the final 3 sessions (retest scores) to the sessions with the same RPE scale from the first 9 sessions (test scores).

#### Data Analyses

To determine absolute agreement between the facial RPE scales and CR10, the RPE scores of the FCR5 scale were multiplied by 2. Workout sessions were excluded from analysis when RPE was missing (e.g., not entered) or not provided on the (subsequent) day of the workout. To calculate the relationship between RPE scores and average heart rate, HR data were synchronized with the workouts using start and end times, after which the HR was cubically interpolated and low-pass filtered (bidirectional second-order Butterworth 2 Hz cutoff filter). For this analysis, data points were removed when RPE or HR was missing or when the duration of the HR data was less than 75% of the duration of the workout. For the reliability analysis, only subjects who completed the 3 final workouts were included (*n* = 42). Finally, session RPE (sRPE) and Edward's TRIMP (eTRIMP) scores were correlated as measures of internal training load: sRPE was determined by multiplying workout duration with the RPE score (17) (based on CR10, FCR10 or FCR5), and eTRIMP scores were calculated based on the time spent in each of the 5 HR zones (10). Data preparation was conducted using Python (version 3.9.7) and R (version 4.0.0).

### Statistical Analyses

To evaluate criterion validity of the facial RPE scales, absolute agreement between FCR5 or FCR10 and CR10 was quantified using intra-class correlations (ICC(A, 1) (20)) and Bland-Altman plots. In addition, Spearman’s correlations were examined between RPE scores and average heart rate. For reliability, test-retest agreement was evaluated based on ICC(A, 1). To evaluate the use of facial RPE scales for internal training load monitoring, Spearman’s correlations were determined between sRPE and eTRIMP scores. Correlation coefficients were interpreted according to Evans (12), with coefficients <0.20, <0.40, <0.60, <0.80, and >0.80 representing very weak, weak, moderate, strong, and very strong correlations. ICC values were interpreted according to Koo and Li (20), with ICC values < 0.50, <0.75, <0.90, and >0.90 representing poor, moderate, good, and excellent agreement, respectively. Group differences in baseline characteristics were assessed between UT, RT, and T subjects using one-way analysis of variance tests, and Bonferroni’s post hoc tests were used to localize differences. Findings were considered to be significant if *p* < 0.05. Statistical Analyses were conducted in R (version 4.0.0).

## Results

### Exercise Groups

Subjects were divided into 3 groups based on the peak power per kilogram they achieved during the maximal incremental test, for male and female athletes. Peak power differed significantly between UT subjects (2.6 ± 0.5; range females: 1.25–2.99; range males: 3.06–3.25), RT subjects (3.8 ± 0.4; range females: 3.10–3.78; range males: 3.81–4.50), and T subjects (4.4 ± 0.4; range females: 3.82–4.64; range males: 4.62–4.81) (see also Table 1).

### Criterion Validity Using CR10 as Golden Standard

**Figure 2.**
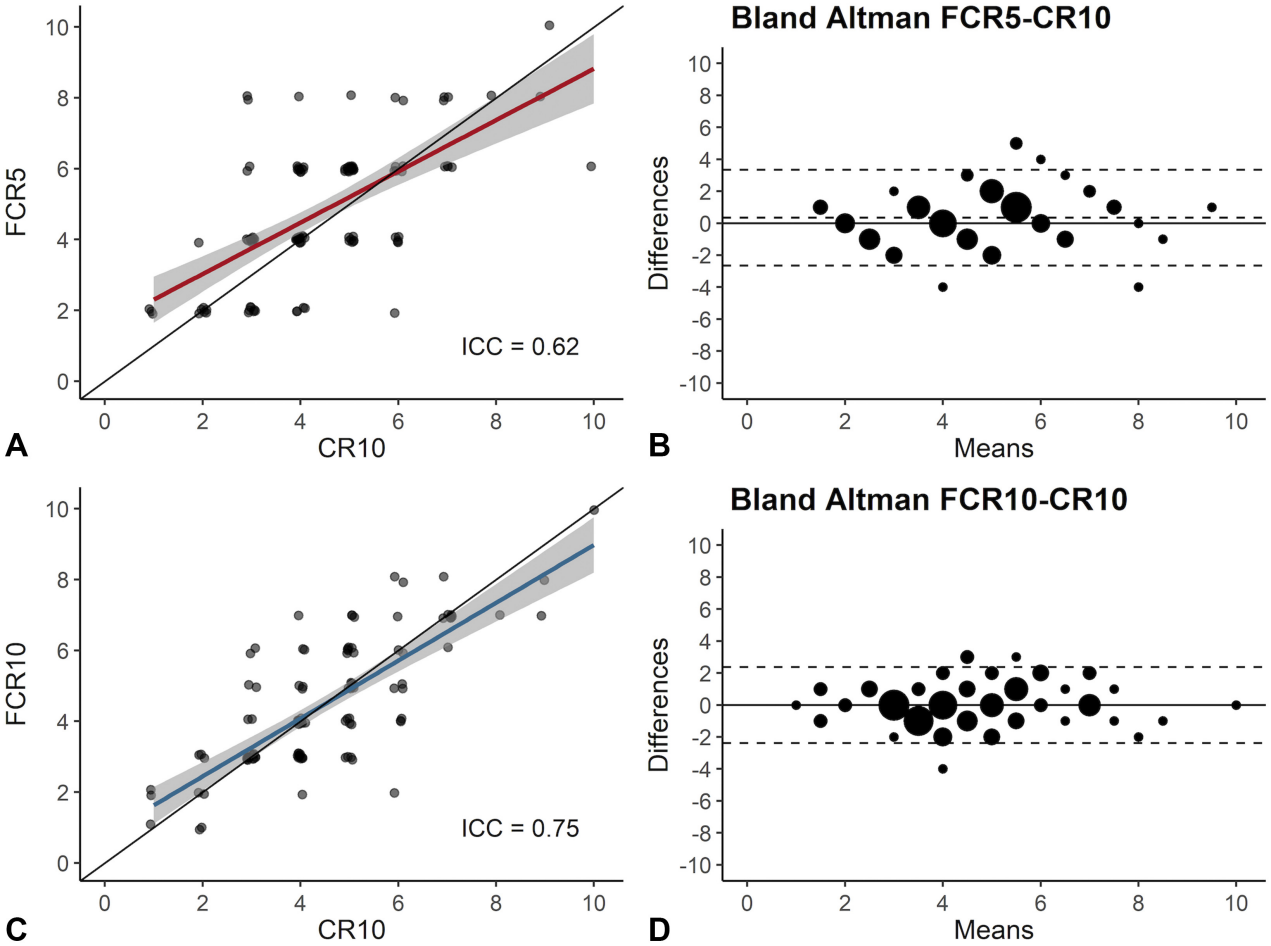
Validity based on absolute agreement between facial RPE scales and CR10, which was used as a golden standard for perceived exertion. Validity was evaluated using intraclass correlations and regression lines (A and C) as well as Bland-Altman plots (B and D), for FCR5 (*n* = 109) and FCR10 (*n* = 109), respectively. For panels A and C, a small jitter was applied to distinguish overlapping RPE values. FCR5: 5-point facial category-ratio RPE scale; FCR10: 10-point facial category-ratio RPE scale; CR10: 10-point category-ratio RPE scale; ICC = intraclass correlation based on absolute agreement using single measures. RPE = rating of perceived exertion.

Criterion validity of FCR5 and FCR10 was evaluated using CR10 as a golden standard (Figure 2 and Table 2), demonstrating moderate absolute agreement between FCR5 and CR10 and good agreement between FCR10 and CR10. Bland-Altman plots showed a significant bias for higher RPE scores with FCR5 (bias = 0.35, CI_95%_ = 0.06–0.64, *p* = 0.019) but not with FCR10 (bias = −0.009, CI_95%_ = −0.24 to 0.22, *p* = 0.937). Differences in validity were observed between UT, RT, and T individuals (Table 2). FCR5 and FCR10 showed poor validity in UT individuals but moderate (FCR5) to good (FCR10) validity in RT and T individuals.

**Table 2:** Validity based on absolute agreement between facial RPE scales and CR10.*

Group	FCR5			FCR10		
	n	ICC (95%CI)	*p*	n	ICC (95%CI)	*p*
All	109	0.62 (0.49–0.72)	<0.001	109	0.75 (0.66–0.83)	<0.001
Untrained	38	0.23 (−0.06–0.49)	0.064	39	0.44 (0.15–0.66)	0.002
Recreational	44	0.72 (0.54–0.84)	<0.001	42	0.80 (0.66–0.89)	<0.001
Trained	27	0.74 (0.51–0.87)	<0.001	28	0.85 (0.71–0.93)	<0.001

* FCR5 = 5-point facial category-ratio RPE scale; FCR10 = 10-point facial category-ratio RPE scale; CR10 = 10-point category-ratio RPE scale; ICC = intraclass correlation based on absolute agreement using single measures.

### Criterion Validity Using Average Heart Rate as Criterion Measure

**Figure 3.**
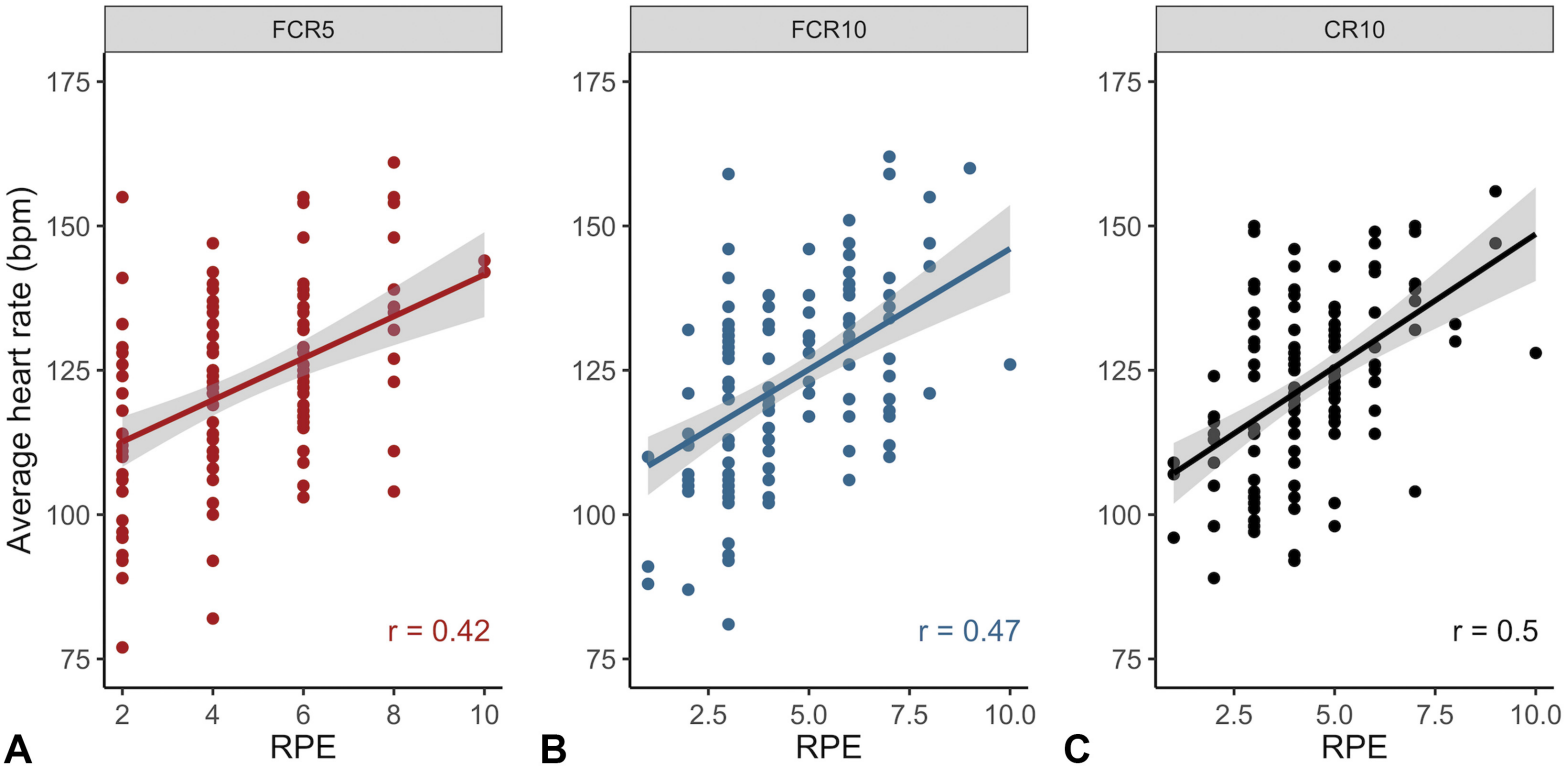
Validity based on the association between average heart rate and RPE scores of FCR5, FCR10, and CR10. Validity was evaluated using Spearman’s correlation coefficients between RPE scores on FCR5 (A), FCR10 (B), or CR10 (C) scale and average heart rate during the workout (FCR5: *n* = 122, FCR10: *n* = 126, CR10: *n* = 123). FCR5: 5-point facial category-ratio RPE scale; FCR10: 10-point facial category-ratio RPE scale; CR10: 10-point category-ratio RPE scale. RPE = rating of perceived exertion.

Validity was also assessed using the average heart rate during workouts (Figure 3). FCR10 and CR10 showed similar correlations with HR, but somewhat weaker correlations were observed for FCR5. Within the groups, correlation coefficients were moderate to strong in RT and T subjects (*p* ≤ 0.02) but low in UT subjects (*p* ≥ 0.51).

### Reliability

**Figure 4.**
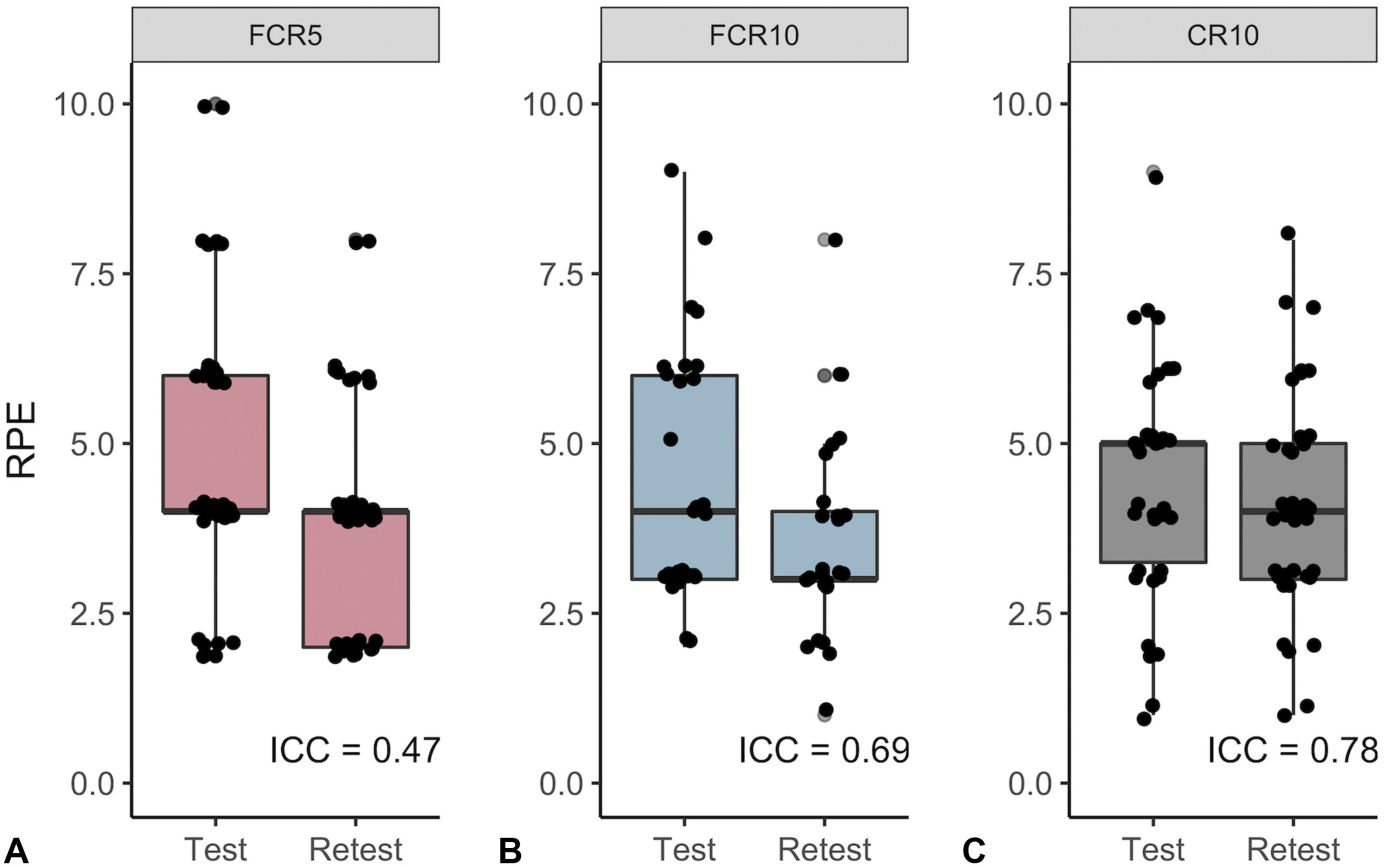
Reliability of FCR5, FCR10, and CR10 was assessed based on absolute agreement between test-retest scores. Reliability was evaluated using intraclass correlations based on absolute agreement and single measures (FCR5: *n* = 38, FCR10: *n* = 22, CR10: *n* = 38). FCR5: 5-point facial category-ratio RPE scale; FCR10: 10-point facial category-ratio RPE scale; CR10: 10-point category-ratio RPE scale. RPE = rating of perceived exertion.

Reliability of FCR5 and FCR10 was determined for the home-based workouts and compared with the reliability of the CR10 (Figure 4). Reliability was poor for FCR5 (ICC = 0.47, *p* = 0.006), moderate for FCR10 (ICC = 0.69, *p* < 0.001), and good for CR10 (ICC = 0.78, *p* < 0.001). In UT, reliability was poor for all RPE scales (ICC ≤ 0.43, *p* ≥ 0.09). However, reliability was higher in both RT and T subjects, which was rather poor for FCR5 (ICC = 0.51, *p* = 0.006), moderate for FCR10 (ICC = 0.74, *p* < 0.001), and excellent for CR10 (ICC = 0.92, *p* < 0.001).

### Training Load Monitoring

We evaluated whether FCR5 and FCR10 could be used for internal training load monitoring of home-based workouts. On average, sRPE was 125 ± 62 and eTRIMP was 62 ± 27. The relationship between sRPE and eTRIMP was moderate to strong and revealed similar correlation coefficients for FCR5 (*r* = 0.60, *p* < 0.001, *n* = 149), FCR10 (*r* = 0.52, *p* < 0.001, *n* = 137), and CR10 (*r* = 0.60, *p* < 0.001, *n* = 147). In both RT and T subjects, this association between sRPE and eTRIMP was stronger, and it also revealed similar correlation coefficients for FCR5 (*r* = 0.72, *p* < 0.001, *n* = 104), FCR10 (*r* = 0.70, *p* < 0.001, *n* = 88), and CR10 (*r* = 0.74, *p* < 0.001, *n* = 90).

### User Experience

From a user-experience perspective, subjects revealed similar preference for each of the three scales (*n* = 39): 36% of the subjects favored FCR5, 33% the FCR10 scale, and 31% CR10. Moreover, most subjects preferred a 10-point scale (62%) as opposed to a 5-point scale (36%), and 3% had no preference. Most subjects favored a facial RPE scale (46%) as opposed to a scale without smileys (21%), and 33% had no preference. Therefore, the FCR10 scale seems to provide the best user experience for the subjects.

## Discussion

The main aim of this study was to evaluate whether a 5-point and 10-point facial RPE scale (FCR5 and FCR10, respectively) could validly and reliably capture exercise intensity in a real-life setting. The CR10 scale was used as a golden standard (14). In general, the FCR5 showed moderate validity and poor reliability when capturing perceived exertion during home-based exercise sessions, whereas FCR10 demonstrated good validity and moderate reliability. However, differences in validity and reliability of the facial scales were found when used by different groups. More specifically, validity and reliability scores were moderate to good in RT and T subjects but poor when used by UT subjects. With respect to internal training load, based on FCR5 and FCR10, sRPE scores were strongly related to eTRIMP in RT and T subjects. User experience was best supported by FCR10. In summary, findings indicate that the facial FCR10 scale—and not the FCR5 scale—is appropriate for capturing perceived exertion and quantifying internal training load in RT and T subjects.

To the best of our knowledge, this study is the first to assess validity of facial RPE scales based on absolute agreement with RPE values from the CR10 scale as a golden standard (1,2). One previous study compared findings from a facial RPE scale and CR10 but only by comparing their correlation coefficients with secondary physiological measures (e.g., workload and heart rate (5)). Statistical measures of agreement, rather than correlation, provide evidence as to whether facial RPE scales can be used interchangeably with the golden standard CR10 scale. Moreover, this study is the first to measure the reliability of facial RPE scales. The reliability of both the FCR5 and FCR10 scale were lower than the reliability of the CR10 scale. However, the reported reliability scores of FCR10 and CR10 were similar or higher than previously reported reliability scores of the CR10 scale for cycling exercises (ICC = 0.75–0.77 and *r*^2^ = 0.78 (18,30)) or Australian Football sessions (ICC = 0.66 (24)) in (recreational) athletes. From these results, it can be concluded that FCR10—but not FCR5—demonstrated sufficient validity and reliability to capture exercise intensity, even in a real-life setting.

Validity was also assessed by correlating RPE scores to an objective physiological marker (4,17). Using average heart rate as criterion measure, we observed very similar correlation coefficients between FCR10 and CR10 and a somewhat lower correlation coefficient for FCR5 in healthy adults. Previous observations in young adults also showed that the 10-point facial RPE scale and CR10 revealed similar correlation coefficients with heart rate (5). Correlation coefficients of FCR5, FCR10, and CR10 indicated moderate to strong relationships, which is comparable to the correlation coefficients for a 6-point RPE scale in older adults and patients with arterial fibrillation (22), but lower than the very strong associations with heart rate observed for a 6-point RPE scale in healthy adults (21) and 10-point facial RPE scale in children and young adults (5). This could be explained because these studies (5,21,22) were performed in a laboratory-based setting or evaluated RPE scores at a particular moment throughout incremental exercise rather than providing a global RPE score for the entire workout. Our correlation coefficients based on global RPE scores were very similar to the correlation coefficients that have previously been reported for global RPE scores of the golden standard CR10 (1,4,18,24). Interestingly, correlation coefficients tended to be lower with a larger sample size (4), demonstrating very strong correlations in only 14 recreationally trained individuals (*r* = 0.76–0.86 (1,18)), strong correlations in 21 Australian football players (*r* = 0.66 (24)), and moderate correlations in a pooled group of 514 individuals (*r* = 0.47 (4)) as well as in our sample of 49 individuals (*r* = 0.50 for CR10, Figure 3). This implies that validation studies should include sufficiently large samples. In brief, the associations between RPE values and heart rate as an objective physiological marker were similar for the FCR10 scale and the golden standard CR10 scale.

One important finding was that validity and reliability of the facial scales were different for UT individuals compared with RT and T individuals. In particular, the validity and reliability of the facial RPE scales were moderate to good when used by RT and T subjects but low when used by UT subjects. Furthermore, for UT, no relationship was found between RPE values and average HR, independent of which RPE scale was used. These observations seem to be in line with previous literature, demonstrating that the average correlation coefficients between RPE values and heart rate tended to be lower in sedentary subjects (*r* = 0.41 (4)) compared with active and highly fit subjects (*r* = 0.60–0.61 (4)). One explanation of this difference could be that UT individuals may experience difficulties with properly evaluating their psychophysiological exertion (as they do not exercise regularly) or may require additional familiarization to better understand the RPE scales. Still, the lower validity and reliability in UT subjects could not be counteracted when using the facial RPE scales that are supposedly easier to understand. Therefore, it remains to be determined how RPE scales could be optimally used to capture the perceived exertion of UT people.

The session-RPE method has been proposed as a simple, noninvasive, and inexpensive method for training load monitoring (17). Prior studies have shown that these training load indices can be useful for evaluating overtraining, illness, or injuries (9,16,19,23). In this study, we observed that RPE scores for the same workout were somewhat higher for FCR5 compared with CR10 (+0.35 AU) but similar for FCR10 and CR10 (−0.01 AU). Considering RPE scores as well as the duration of the workout, sRPE values were strongly related to the heart-rate based eTRIMP scores in RT and T subjects, irrespective of the used RPE scale (*r* ≥ 0.70). These correlation coefficients are in line with previous observations based on the CR10 scale in sports such as basketball, diving, football, karate, rowing, soccer, swimming, taekwondo, tennis, and water polo (17). Because we observed poor validity and reliability for UT, we discourage the use of facial RPE scales for training monitoring in this group, as it should first be known how these scales can be optimally used in this population. However, in RT and T subjects, our findings suggest that training load monitoring can be easily accomplished using the FCR10 scale but not using FCR5 because of its low(er) validity and reliability.

This study was conducted in a real-life context with home-based training sessions to increase the ecological validity and to generalize the results into practice. Because of this design, some limitations are addressed. First, because subjects performed their workouts at home, it could not be actively monitored how precisely subjects followed the video instructions for every workout. Although we intentionally repeated the same light, medium, and hard workouts for every RPE scale, a potential difference in adherence to the instructions might have reduced the ICC values for validity and reliability. Nonetheless, reliability of the golden standard CR10 was excellent (ICC = 0.92) in RT and T subjects, even for home-based workouts. Second, we experienced that the average heart rate during the whole-body workouts was not very high (67 ± 8% of maximal heart rate), likely because workouts contained a combination of strength-based and aerobic exercises and rest was included between the exercises. Presumably, correlation coefficients between RPE and heart rate may be higher when only well-defined aerobic exercises such as cycling at a fixed power output are considered (1,18). Third, we used a global rating for the entire exercise session, in accordance with previous instructions (14). The advantage of such a procedure is that this provides additional information on training load monitoring (based on sRPE scores). However, validity and reliability of global RPE scores may be different from how subjects perceive exertion at a particular moment during exercise. Such a momentary RPE score is often used when analyzing pacing behavior in competitive exercise of athletes (6) up to patients (29) or in other settings of exercise regulation (11). Although this was not an aim of this study, future studies may assess the absolute agreement and reliability for momentary RPE scores based on facial RPE scales.

Researchers, coaches, strength and conditioning professionals, and digital sports platforms are encouraged to incorporate the FCR10 scale instead of the FCR5 scale to assess perceived exertion and internal training load of recreationally trained and trained individuals in a real-life setting. Criterion validity was moderate and reliability was rather poor for FCR5, whereas validity was good and reliability was moderate for FCR10 in recreationally trained and trained individuals. In addition, user experience was best supported by the FCR10 scale. Why validity and reliability were lower in untrained subjects remain an unsolved question to be answered.

**Practical Applications**

Nowadays, digital sports platforms incorporate facial RPE scales for monitoring exercise intensities and training load. This study was the first to investigate validity and reliability of 2 facial RPE scales in a real-life setting with a high ecological validity. Although the use of a simple 5-point facial RPE scale seems attractive, present findings discourage implementation of FCR5 because of its low(er) criterion validity and reliability. Instead, implementation of the more valid and reliable FCR10 scale is recommended for strength and conditioning professionals and researchers, at least in recreationally trained and trained individuals. Similar to CR10, the FCR10 has shown to be useful for monitoring the internal training load in these individuals. In addition, FCR10 best supports the user experience. Our results indicate that none of the RPE scales had sufficient validity and reproducibility to assess perceived exertion in untrained individuals.

## References

[1] Arney BE Glover R Fusco A . Comparison of RPE (rating of perceived exertion) scales for session RPE. Int J Sports Physiol Perform 14: 994–996, 2019. 30569764 10.1123/ijspp.2018-0637

[2] Borg E Kaijser L. A comparison between three rating scales for perceived exertion and two different work tests. Scand J Med Sci Sports 16: 57–69, 2006. 16430682 10.1111/j.1600-0838.2005.00448.x

[3] Borg G. Borg's Perceived Exertion and Pain Scales. Champaign, IL: Human Kinetics, 1998.

[4] Chen MJ Fan X Moe ST. Criterion-related validity of the Borg ratings of perceived exertion scale in healthy individuals: A meta-analysis. J Sports Sci 20: 873–899, 2002. 12430990 10.1080/026404102320761787

[5] Chen YL Chiou WK Tzeng YT Lu CY Chen S-C. A rating of perceived exertion scale using facial expressions for conveying exercise intensity for children and young adults. J Sci Med Sport 2017: 66–69, 2017. 10.1016/j.jsams.2016.05.009 27267301

[6] De Koning JJ Foster C Bakkum A . Regulation of pacing strategy during athletic competition. PLoS One 6: e15863, 2011. 21283744 10.1371/journal.pone.0015863 PMC3024328

[7] De Pauw K Roelands B Cheung SS . Guidelines to classify subject groups in sport-science research. Int J Sports Physiol Perform 8: 111–122, 2013. 23428482 10.1123/ijspp.8.2.111

[8] Decroix L De Pauw K Foster C Meeusen R. Guidelines to classify female subject groups in sport-science research. Int J Sports Physiol Perform 11: 204–213, 2016. 26182438 10.1123/ijspp.2015-0153

[9] De Leeuw AW van der Zwaard S van Baar R Knobbe A. Personalized machine learning approach to injury monitoring in elite volleyball players. Eur J Sport Sci: 22: 511–520, 2022. 33568023 10.1080/17461391.2021.1887369

[10] Edwards S. Heart Rate Monitor Book. Port Washington, NY: Sacramento, CA: Polar Electro Inc, 1993.

[11] Eston R. Use of ratings of perceived exertion in sports. Int J Sports Physiol Perform 7: 175–182, 2012. 22634967 10.1123/ijspp.7.2.175

[12] Evans JD. Straightforward Statistics for the Behavioral Sciences. Belmont, CA: Thomson Brooks/Cole Publishing Co, 1996.

[13] Fitchannel. Available at: https://www.fitchannel.com/. Accessed December 3, 2021.

[14] Foster C Florhaug JA Franklin J . A new approach to monitoring exercise training. J Strength Cond Res 15: 109–115, 2001. 11708692

[15] Foster C Boullosa D McGuigan M . 25 years of session rating of perceived exertion: Historical perspective and development. Int J Sports Physiol Perform 16: 612–621, 2021. 33508782 10.1123/ijspp.2020-0599

[16] Foster C. Monitoring training in athletes with reference to overtraining syndrome. Med Sci Sports Exerc 30: 1164–1168, 1998. 9662690 10.1097/00005768-199807000-00023

[17] Haddad M Stylianides G Djaoui L Dellal A Chamari K. Session-RPE method for training load monitoring: Validity, ecological usefulness, and influencing factors. Front Neurosci 11: 612, 2017. 29163016 10.3389/fnins.2017.00612 PMC5673663

[18] Herman L Foster C Maher MA Mikat RP Porcari JP. Validity and reliability of the session RPE method for monitoring exercise training intensity. South Afr J Sports Med 18: 14–17, 2006.

[19] Jones CM Griffiths PC Mellalieu SD. Training load and fatigue marker associations with injury and illness: A systematic review of longitudinal studies. Sports Med 47: 943–974, 2017. 27677917 10.1007/s40279-016-0619-5 PMC5394138

[20] Koo TK Li MY. A guideline of selecting and reporting intraclass correlation coefficients for reliability research. J Chiropr Med 15: 155–163, 2016. 27330520 10.1016/j.jcm.2016.02.012 PMC4913118

[21] Morishita S Tsubaki A Nashimoto S Fu JB Onishi H. Face scale rating of perceived exertion during cardiopulmonary exercise test. BMJ Open Sport Exerc Med 4: e000474, 2018. 10.1136/bmjsem-2018-000474 PMC6307607 30622732

[22] Nashimoto S Morishita S Iida S Hotta K Tsubaki A. Relationship between the face scale for rating of perceived exertion and physiological parameters in older adults and patients with atrial fibrillation. Physiol Rep 9: e14759, 2021. 33650814 10.14814/phy2.14759 PMC7923560

[23] Putlur P Foster C Miskowski JA . Alteration of immune function in women collegiate soccer players and college students. J Sports Sci Med 3: 234–243, 2004. 24624008 PMC3938062

[24] Scott TJ Black CR Quinn J Coutts AJ. Validity and reliability of the session-RPE method for quantifying training in Australian football: A comparison of the CR10 and CR100 scales. J Strength Cond Res 27: 270–276, 2013. 22450253 10.1519/JSC.0b013e3182541d2e

[25] Smartabase. Available at: https://www.fusionsport.com/smartabase/. Accessed December 3, 2021.

[26] Sport Data Valley. Sport Data Valley. Available at: https://info.sportdatavalley.nl/. Accessed December 3, 2021.

[27] Strava. Available at: https://www.strava.com/. Accessed December 3, 2021.

[28] TrainingPeaks. Available at: https://www.trainingpeaks.com/. Accessed December 3, 2021.

[29] Van der Zwaard S Rougoor G van Kasteel PY . Graded exercise testing versus simulated competition exercise in trained older males. J Cardiopulm Rehabil Prev 35: 423–430, 2015. 26252345 10.1097/HCR.0000000000000135

[30] Wallace LK Slattery KM Impellizzeri FM Coutts AJ. Establishing the criterion validity and reliability of common methods for quantifying training load. J Strength Cond Res 28: 2330–2337, 2014. 24662229 10.1519/JSC.0000000000000416

